# “Forget to Whom You Have Told This Proverb”: Directed Forgetting of Destination Memory in Alzheimer's Disease

**DOI:** 10.1155/2015/215971

**Published:** 2015-03-30

**Authors:** Mohamad El Haj, Marie-Charlotte Gandolphe, Philippe Allain, Luciano Fasotti, Pascal Antoine

**Affiliations:** ^1^SCALab UMR CNRS 9193, University of Lille, 59653 Villeneuve d'Ascq Cedex, France; ^2^Laboratoire de Psychologie des Pays de la Loire (EA 4638), LUNAM Université, Université d'Angers, 49000 Angers, France; ^3^Centre Mémoire de Ressources et de Recherches, CHU Angers, 49000 Angers, France; ^4^Centre National de Référence des Maladies Neurogénétiques, CHU Angers, 49000 Angers, France; ^5^Donders Institute for Brain, Cognition and Behaviour, Radboud University Nijmegen, 6500 HP Nijmegen, Netherlands

## Abstract

Destination memory is the ability to remember the receiver of transmitted information. By means of a destination memory directed forgetting task, we investigated whether participants with Alzheimer's Disease (AD) were able to suppress irrelevant information in destination memory. Twenty-six AD participants and 30 healthy elderly subjects were asked to tell 10 different proverbs to 10 different celebrities (List 1). Afterwards, half of the participants were instructed to forget the destinations (i.e., the celebrities) whereas the other half were asked to keep them in mind. After telling 10 other proverbs to 10 other celebrities (List 2), participants were asked to read numbers aloud. Subsequently, all the participants were asked to remember the destinations of List 1 and List 2, regardless of the forget or remember instructions. The results show similar destination memory in AD participants who were asked to forget the destinations of List 1 and those who were asked to retain them. These findings are attributed to inhibitory deficits, by which AD participants have difficulties to suppress irrelevant information in destination memory.

## 1. Introduction

Episodic memory decline has been proposed as the cognitive hallmark of Alzheimer's Disease (AD) [1–6]. This decline can be related to inhibitory dysfunction. Since inhibitory weakness, as observed in aging, is argued to saturate memory with too much information, this may result in competition between appropriate and inappropriate information at the moment of retrieval [7, 8]. In line with this idea, a compromised inhibitory ability was observed in several studies with subjects suffering from AD. In these studies, it was shown that AD participants have difficulties in suppressing irrelevant information in memory. This finding was investigated with the directed forgetting method [9–11].

In its conventional configuration, the directed forgetting list method requires the processing of two lists of words (i.e., List 1 + List 2) [12–17]. Subjects are typically asked to retain the words of List 1, after which they are instructed either to continue remembering or to forget the words of this list. Subsequently, participants are asked to retain the words of List 2. Finally, in a recall test, they are asked to recall all of the words of both the lists, regardless of the previous forget or remember instructions. Two main effects of the directed forgetting method are described: a cost and a benefit effect. The directed forgetting cost refers to the observation that participants with the forget instruction usually show poorer memory for the items of List 1 than the remember participants. The directed forgetting benefit refers to the finding that sometimes the subjects with the forget instruction show better memory for the items of List 2 than the remember participants. Although it has also been found that List 1 cost may occur without List 2 benefit, both directed forgetting effects have been attributed to retrieval inhibition [12–20]. According to the retrieval inhibition explanation, the forget instruction induces a suppression of the List 1 words, making them less accessible. The same instruction also reduces the proactive interference of these words in List 2, thereby improving retrieval of List 2.

Using the directed forgetting method, several authors were able to show a compromised retrieval inhibition in subjects with AD. Collette et al. [9], for instance, designed a working memory directed forgetting task in which AD participants were asked to retrieve one trigram of consonants after a “to be forgotten” instruction was given. The results of this study showed that AD participants had difficulties in suppressing no-longer relevant information in working memory. A finding that was extended to autobiographical memory, or the memory for information related to the self [21, 22]. In the same domain, El Haj et al. [11] asked AD participants to generate autobiographical memories (i.e., List 1). Afterwards, half of the participants were told that these memories were no more relevant to the experiment (i.e., forget participants), whereas the other half was asked to keep these memories in mind (i.e., remember participants). Subsequently, all the participants were asked to generate other autobiographical memories (i.e., List 2), and in a later recall test, they were asked to reconstruct all the memories regardless of previously given forget or remember instructions (i.e., List 1 + List 2). The results showed no effect of the forget instruction, since similar recall performances for List 1 were seen in the remember and the forget AD participants. The absence of directed forgetting costs in the AD subjects was attributed by El Haj et al. [11] to an impairment of autobiographical memory suppression skills.

Compromised suppression ability in AD was also extended to source memory, or the ability to remember the context in which an event has occurred [23, 24]. This assumption was tested in a study in which AD participants were asked to remember the source of presentation of items that were presented by an experimenter with a black- or white-gloved hand (i.e., List 1) [10]. Afterwards, half of the participants were instructed to forget the source of List 1, whereas the other half was asked to keep it in mind. Subsequently, all the participants were asked to retain the source of presentation of a second List of items (i.e., List 2), and in a later recall test, they were asked to remember the source of presentation of items of List 1 and List 2, regardless of the forget or remember instructions. The results showed no effect of forget instruction, since no differences were visible between the remember and the forget AD participants on retrieving the sources of List 1 items. Taken together, empirical evidence suggests substantial difficulties in AD participants when they are asked to suppress no-longer relevant information in working memory [9], source memory [10], and autobiographical memory [11]. The present paper is aimed at investigating these findings in destination memory.

Destination memory is the ability to remember the receiver of transmitted information (e.g., did I tell* you *about the weekend?) [25–28]. This ability can be considered as an element of the episodic memory system, since destination recall allows the specification of the context in which an episodic event has occurred [25]. In line with this view, destination memory was found to be compromised in AD subjects [29, 30], a deterioration that was found to be related to episodic memory decline [31]. Destination memory decline in AD may result in redundancy, or the tendency to repeat the same information to the same person [25, 32]. Such a distortion may involve annoying issues, such as a multiplication of inferences and a reduction of the amount of stored new information. These consequences may attenuate the amount of social interaction between individuals with AD and their environment. AD-related destination memory deterioration may also be related to inhibitory decline, since significant correlations were observed in a previous study between destination memory abilities and performance on the Stroop task [29]. However, these correlations were found in a composite sample of younger participants, healthy elderly, and AD participants, without specifically investigating AD participants.

To summarize, several studies suggest difficulties in AD participants when suppressing no-longer relevant information in working memory [9], source memory [10], and autobiographical memory [11]. The present paper is aimed at investigating these issues specifically with destination memory. Therefore, AD participants and healthy older adults were asked to tell proverbs to celebrities and either to forget or to continue remembering the destination (i.e., List 1). Subsequently, all participants had to tell other proverbs to other celebrities (i.e., List 2). This is to retrieve, on a later recognition test, the destinations to which the proverbs of List 1 and List 2 were conveyed, regardless of the forget or remember instructions. Similarly to the studies that had not shown forget instruction effects on AD patients [10, 11], we expected similar destination memory performances on List 1 in forget and remember conditions with AD participants. In order to further elucidate the relationship between inhibition and the ability to forget or remember destinations for List 1, we expected significant correlations between the latter ability and performance on the Stroop task.

## 2. Method

### 2.1. Participants

The study included 26 participants with a clinical diagnosis of probable mild AD (18 women and 8 men, M age = 73.38 years, SD = 6.86; M years of formal education = 8.77, and SD = 2.73) and 30 healthy older adults (21 women and 9 men, M age = 72.30 years, SD = 7.40, M years of formal education = 9.87, and SD = 2.80). The AD participants were recruited from local retirement homes. They were diagnosed with probable AD dementia based on the NINCDS-ADRDA clinical criteria [1]. Controls were often spouses or companions of AD participants and were living independently at home. The two samples were matched according to sex [*X* ^2^ (1, *N* = 56) = .004, *P* > .10], age [*t*(54) = .54, *P* > .10], and educational level [*t*(54) = 1.48, *P* > .10].

All participants consented freely to participate in this study and were given the opportunity to withdraw whenever they wished. Exclusion criteria were significant neurological or psychiatric illness and major visual or auditory acuity difficulties that could prevent adequate assessment. The clinical and cognitive characteristics of all the participants were assessed with a comprehensive battery of neuropsychological tests and questionnaires, detailed below.

### 2.2. Procedures

#### 2.2.1. Clinical and Cognitive Assessment

We used tests and questionnaires tapping general cognitive functioning, episodic memory, inhibition, anxiety, and depression. General cognitive functioning was assessed with the Mini Mental State Examination [33], with a maximal score of 30 points. Episodic memory was evaluated with the Grober and Buschke [34] task. Participants had to retain 16 words, and, after immediate cued recall, they went into a distraction phase, during which they had to count backwards from 374 during 20 s. The distraction phase was immediately followed by 2 minutes of free recall and the score (with a maximum of 16) of this phase is considered a measure of episodic recall. Inhibition was assessed with the Stroop task, involving three conditions (i.e., word reading, color naming, and color-word interference). In the word reading condition, participants had to read, as fast as they could, words of colors printed in black ink. In the color naming condition, participants had to name, as fast as possible, the color of rectangles. In the interference condition, participants had to name, also as fast as possible, the color of color-words printed in an incongruous color (e.g., the word “green” written in red). The inhibition score referred to completion time for the interference condition minus the average completion time for the word reading and color naming conditions. For the assessment of anxiety and depression, we used the Hospital Anxiety and Depression Scale [35]. This scale consists of seven items assessing anxiety and seven items examining depression. Items were scored by the participants on a four-point scale from 0 (not present) to 3 (considerable) and the cut-off score for anxiety and depression was set at > 10/21 points. The scores on clinical and cognitive tasks are summarized in Table 1.

#### 2.2.2. Directed Forgetting Task

**Figure 1:**
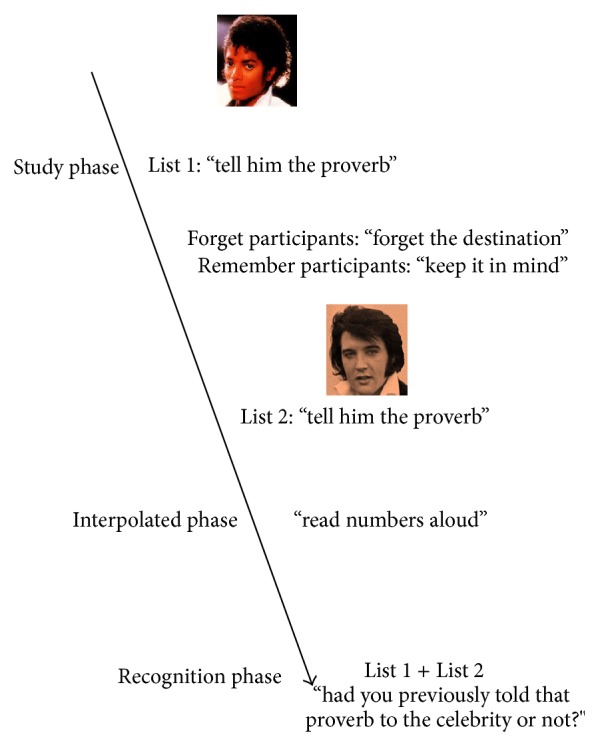
In the destination memory directed forgetting task, participants had to tell 10 different proverbs to 10 different celebrities (List 1). Afterward, half the participants was instructed to forget this List whereas the other half was asked to keep it in mind. After telling 10 other proverbs to 10 other celebrities (List 2), participants had to read numbers aloud. Subsequently, all the participants were asked to remember the destinations in List 1 and List 2, regardless of the forget or remember instructions.* Note. *Celebrities' images are covered by creative commons copyright.

As illustrated in Figure 1, the directed forgetting task included a study phase, an interpolated phase, and a recognition phase. The study phase included two Lists, List 1 and List 2, each one including 10 trials. Each trial began with a proverb presented in black Times New Roman 48-point font, printed below a (16 × 16 cm) colored picture of a celebrity face; both the proverb and celebrity face were presented on a white A4 paper. Participants were asked to tell the proverb to the celebrity with no time limit. Successively, they were presented with another proverb and celebrity until they told 10 different proverbs to 10 different celebrities. In this study phase, participants were informed that their memory for the association between proverbs and celebrities would be tested in a later session. However, at the end of List 1 presentation, half of the participants (i.e., the forget participants) were told the following: “in fact, the just presented list was only for practice. You have to forget the destinations of the previous proverbs, and now you have to remember the destinations of another 10 proverbs, this for a later recall test.” The other half of the participants (i.e., the remember participants) were told that after completing List 1 they had to prepare themselves for a second list to be recalled later. Assignment to the forget or remember group was randomized. After the presentation of List 2, all participants were asked to participate in an interpolated activity that consisted of reading strings of three digits aloud for one min. The interpolated phase was immediately followed by the recognition phase in which the participants had to retrieve the destinations of List 1 and List 2, regardless of the forget or remember instructions. To this aim, the experimenter presented the previously exposed 20 proverbs and faces: 10 proverb-face pairs were intact, and the remaining 10 proverb-face associations were reshuffled. Pairs were presented one at a time, with the proverb being under the face. For each pair, the participants had to decide, with no time limit, whether they had previously told that proverb to that celebrity or not. Recognition performance was the proportion of correct “yes” responses (i.e., when participants correctly remembered that they had previously told that proverb to that celebrity) + correct “no” responses (i.e., when participants correctly remembered that they had not told that proverb to that celebrity) (for the same scoring method, see, [36]).

This destination memory directed forgetting task was adopted from studies assessing destination memory in normal aging and pathological aging [27, 29–32]. Familiarity of celebrities and proverbs was controlled in a previous work with older adults [32]. During the study and recognition phases, proverb-face pairs correspondence was prerandomized and the correspondence was identical for all participants. During the recognition phase, the experimenter was careful to note the participant's answers on special grids so that right and wrong answers could be easily analyzed.

### 2.3. Results

The recognition scores for List 1 and List 2 are displayed in Table 2. Due to a nonnormal distribution of the data, nonparametric tests were used. We first assessed group effects (AD versus older adults) and then instruction effects (forget versus remember) on mean destination memory scores. Next, we assessed directed forgetting cost by investigating the differences on List 1 between participants who were instructed to forget it (i.e., the forget participants) and those who were instructed to remember it (i.e., the remember participants). Directed forgetting benefit was also assessed by analyzing the differences on List 2 between participants who were instructed to forget List 1 (i.e., the forget participants) and those who were instructed to remember List 1 (i.e., the remember participants). We finally calculated, in each sample, the correlations between destination memory in List 1, destination memory in List 2, and inhibition as evaluated with the Stroop task.

#### 2.3.1. No Directed Forgetting Effects in AD Participants or Healthy Elderly

With regard to general performance, a Mann-Whitney *U* test revealed poorer destination memory in AD participants (M = .37, SD = .22) than in older adults (M = .58, SD = .22) (*Z* = 4.11, *P* < .001, *η* ^2^ = .95), whereas no significant differences were observed between forget participants (AD + older adults) (M = .47, SD = .23) and remember participants (M = .50, SD = .26) (*Z* = .72, *P* > .1, *η* ^2^ = .22). No directed forgetting cost was found for AD participants, since no significant differences could be detected on List 1 between those who were instructed to forget it and those who were instructed to remember it (*Z* = .18, *P* > .1, *η* ^2^ = .22). The same result was found in older adults (*Z* = .21, *P* > .1, *η* ^2^ = .17). No directed forgetting benefit was observed for AD participants, since no significant differences could be found in List 2 between those who were instructed to forget and those who were instructed to remember List 1 (*Z* = .36, *P* > .1, *η* ^2^ = .08). The same result was found in older adults (*Z* = .35, *P* > .1, *η* ^2^ = .17).

It is noteworthy that poorer destination memory (i.e., mean (List 1 + List 2)) was seen in AD participants with the remember instruction in comparison with healthy older adults with the same instruction (*Z* = 2.14, *P* < .05, *η* ^2^ = .21). Poorer destination memory (i.e., mean (List 1 + List 2)) was seen in AD participants with the forget instruction in comparison with healthy older adults with the same instruction (*Z* = 2.69, *P* < .01, *η* ^2^ = .29). Poorer destination memory was seen in AD participants instructed to forget List 1 in comparison with healthy older adults (*Z* = 2.92, *P* < .01, *η* ^2^ = .11). AD participants performed also poorer than older adults when instructed to remember List 1 (*Z* = 2.01, *P* < .05, *η* ^2^ = .10). AD participants instructed to forget List 2 remembered significantly less proverb-face associations than older adults (*Z* = 1.91, *P* = .05, *η* ^2^ = .08). Finally, AD participants instructed to remember List 2 performed worse than older adults with the same instruction (*Z* = 2.03, *P* < .05, *η* ^2^ = .20). No significant differences were seen between (a) AD participants instructed to forget List 1 and those instructed to forget List 2 (*Z* = .61, *P* > .1, *η* ^2^ = .21), (b) AD participants instructed to remember List 1 and those instructed to remember List 2 (*Z* = .04, *P* > .1, *η* ^2^ = .11), (c) older adults instructed to forget List 1 and those instructed to forget List 2 (*Z* = .09, *P* > .1, *η* ^2^ = .04), and (d) older adults instructed to remember List 1 and those instructed to remember List 2 (*Z* = .28, *P* > .1, *η* ^2^ = .04).

#### 2.3.2. Relationship between Performance on List 1 and List 2 and Inhibition

As shown in Table 3, significant correlations were found between destination memory in List 1, destination memory in List 2, and inhibition as evaluated with the Stroop task in AD participants and older adults. The correlations were negative; poor scores on destination memory were significantly correlated with higher completion times (i.e., poor performance) in the Stroop task.

## 3. Discussion

The aim of the present study was to assess the ability of individuals with AD to suppress irrelevant information in destination memory. Using a destination memory directed forgetting task we found no directed forgetting cost in AD participants, since similar destination memory scores were seen in AD participants asked to forget destinations in List 1 and those asked to retain these destinations. Therefore, AD participants seem to have difficulties suppressing irrelevant information about the destination of their thoughts, sayings, and actions.

Generally speaking, cognitive inhibition refers to the processes that allow the suppression of previously activated schemes, the clearing of irrelevant actions or thoughts from consciousness, and the resistance to interference from potentially attention-capturing information [37–39]. In AD, inhibition decline is considered as an important characteristic of cognitive decline in the early stages of the disease [40–43]. In line with this idea, Amieva et al. [40] found evidence for early compromised inhibitory abilities in AD. This decline was specifically observed for controlled inhibition, as assessed with the Stroop task or the directed forgetting method. Automatic inhibition, as assessed with inhibition of a return paradigm in which participants typically have to attend specific regions of space, seemed less compromised. In the same vein, several studies showed compromised controlled inhibitory ability in AD on the Hayling task in which participants have to complete sentences with unrelated words [9, 41]. Also a study showed AD-related difficulties in a go/no-go task in which participants have to voluntarily inhibit a motor response driven by an external cue [44]. From all these studies, it can be concluded that AD is characterized by a severe impairment of inhibitory mechanisms (for a review, see [40]).

AD-related inhibitory decline can be extended to the ability underlying the inhibition of irrelevant information in memory. With the help of the directed forgetting method, different studies have suggested difficulties in AD participants to suppress irrelevant information in working memory [9], source memory [10], and autobiographical memory [11]. Our study extends these conclusions to destination memory, since our AD participants showed similar destination recognition abilities for the “to-be forgotten” and “to-be remembered” destinations. Failure to suppress irrelevant destination representations as observed in our AD participants can be related to a decline of retrieval inhibition. According to the retrieval inhibition hypothesis [12], the forget instruction triggers inhibitory mechanisms that reduce the accessibility of the to-be forgotten information in memory. Therefore, retrieval inhibition is a form of forgetting whereby irrelevant memories are actively suppressed to the extent that they are no longer available to conscious access (for the same view, see [45]). This view is of compelling interest, since retrieval inhibition may be distinguished from traditional conceptions of forgetting by which memory loss is considered as a gradual deterioration of representation traces over time. AD-related memory decline shows that memory deterioration may be related not only to loss of information over time but also to a failure to suppress no-longer relevant information.

Retrieval inhibition decline is also worth highlighting because it contributes to a better understanding of the mechanisms underlying episodic memory deterioration in AD. Episodic memory impairments in AD have been generally considered as characterized by retrieval deficits. It is likely that these impairments are related to retrieval inhibition decline, by which increased competition between appropriate and inappropriate information takes place at the moment of retrieval, leading to poor recuperation and, consequently, poor memory performance. Retrieval inhibition decline may also be incorporated into a general model of executive dysfunction since inhibition has been considered as a core executive ability [38]. On a social level, difficulties to inhibit destination memory enhance redundancy or the tendency to repeat the same information to the same correspondent. This finding is supported by studies showing a relationship between comprised inhibitory ability in normal aging and off-target verbosity or extended speech that may start out on a topic but quickly becomes prolonged and irrelevant to the main topic [46–50]. This excessive talkativeness is an important feature of social communication in individuals with AD, as these individuals tend to repeat themselves or ask the same question to the same correspondent. A communication bias may be interpreted in terms of difficulties to inhibit the tendency to emit the same information to the same destination.

Our AD participants showed no directed forgetting cost. They also showed no directed forgetting benefits, since similar destination memory performance was observed in participants asked to forget List 2 and those asked to retain it. The latter outcome can be interpreted in terms of difficulties in adaptation to contextual changes. According to the contextual account of directed forgetting, after being asked to forget List 1, forget participants adopt a strategy change by which they develop a more elaborate encoding of List 2 than the remember participants [19, 51]. This contextual adaptation gives rise to a better recall on List 2 in the forget participants than in the remember participants. Following this reasoning, it is likely that the absence of List 2 benefits in our AD participants may be related to difficulties in the adaptation to context changes and the lack of an efficient strategy to process destinations of List 2.

Like AD participants, older adults showed no directed forgetting effects, an outcome that mirrors studies suggesting an impairment of intentional inhibitory processes in episodic memory in normal aging [20, 52]. It is noteworthy that this age-related memory compromising has been widely attributed to inhibitory decline, resulting in competition between appropriate information and inappropriate information at the moment of retrieval [7].

A shortcoming of our paper is the absence of a classic directed forgetting list method [6], in which participants are asked to process word lists rather than destinations. Such a measure might provide further evidence for the assumption that AD patients have difficulties in inhibiting information in episodic memory.

To summarize, episodic memory decline has been widely considered as a hallmark of AD. Our work extends this finding by attributing this decline to inhibitory deficits by which poor suppression abilities lead to competition between appropriate information and inappropriate information during retrieval and, consequently, to poor memory performance. Hence, there is much-felt need to include measures of inhibitory functioning in the clinical assessment of memory problems in AD subjects.

## Figures and Tables

**Table 1:** Cognitive and clinical characteristics of Alzheimer's disease (AD) patients and control participants.

	Task	AD *n* = 26	Older adults *n* = 30
General cognitive functioning	Mini-mental state examination (MMSE)	21.85 (1.46)^***^	28.33 (1.24)
Episodic memory	Grober and Buschke	6.00 (2.36)^***^	10.97 (3.09)
Inhibition	Stroop	61.08 (9.48)^***^	35.95 (12.93)
Anxiety	HADS	8.42 (2.74)^***^	6.53 (2.27)
Depression		10.62 (3.56)^***^	6.65 (2.41)

*Note.* Standard deviations are between brackets; the maximum score on the MMSE was 30 points; the maximum score on the Grober and Buschke task was 16 points; scores on the Stroop task were reaction times; the cut-off score of the HADS (Hospital Anxiety and Depression Scale) was >10/21 points; differences between groups were significant at ^***^ *P* < .001.

**Table 2:** Recognition scores for List 1 and List 2 found in the directed forgetting groups.

	Alzheimer's Disease *n* = 26		Older adults *n* = 30	
	Forget	Remember	Forget	Remember
List 1	.34 (.17)	.37 (.23)	.56 (.22)	.60 (.24)
List 2	.38 (.21)	.40 (.28)	.57 (.24)	.61 (.22)

*Note.* Standard deviations are given between brackets.

**Table 3:** Correlations between destination memory in List 1, destination memory in List 2, and inhibition as evaluated with the Stroop task.

		List 1	List 2	Inhibition
List 1	Alzheimer's Disease subjects	—		
List 2		.65^**^	—	
Inhibition		−.57^**^	−.45^*^	—

List 1	Older adults	—		
List 2		.49^**^	—	
Inhibition		−.54^**^	−.36^*^	—

*Note.* Correlations were significant at ^*^ *P* < .05 and ^**^ *P* < .01.
